# Outbreak of Klebsiella pneumoniae ST11 Resistant To Ceftazidime-Avibactam Producing KPC-31 and the Novel Variant KPC-115 during COVID-19 Pandemic in Argentina

**DOI:** 10.1128/spectrum.03733-22

**Published:** 2022-11-29

**Authors:** Federico Nicola, Daniela Cejas, Francisco González-Espinosa, Silvia Relloso, Fabián Herrera, Pablo Bonvehí, Jorgelina Smayevsky, Roque Figueroa-Espinosa, Gabriel Gutkind, Marcela Radice

**Affiliations:** a Laboratorio de Bacteriología, Micología y Parasitología, Departamento de Análisis Clínicos, Centro de Educación Médica e Investigaciones Clínicas (CEMIC), Buenos Aires, Argentina; b Universidad de Buenos Aires, Facultad de Farmacia y Bioquímica, Instituto de Investigaciones en Bacteriología y Virología Molecular (IBaViM), Buenos Aires, Argentina; c Consejo Nacional de Investigaciones Científicas y Técnicas (CONICET), Buenos Aires, Argentina; d Sección Infectología, Departamento de Medicina Interna, Centro de Educación Médica e Investigaciones Clínicas (CEMIC), Buenos Aires, Argentina; Riverside University Health System, Medical Center–University of California

**Keywords:** KPC carbapenemases, ST11 *Klebsiella pneumoniae*, cettazidime-avibactam resistance, mutant KPC

## Abstract

We describe an outbreak of Klebsiella pneumoniae sequence type 11 (ST11) producing KPC variants resistant to ceftazidime-avibactam. Six patients hospitalized in the intensive care unit (mostly due to critical COVID pneumonia) presented infection or colonization by this bacterium. They had several comorbidities and required mechanical ventilation, central venous catheters, and urinary catheters. All 6 patients had a history of fecal colonization with KPC-producing *Enterobacterales* (KPC-E). Three of them had previous episodes of infection with ceftazidime-avibactam-susceptible KPC-producing K. pneumoniae, which were treated with ceftazidime-avibactam. Several phenotypic methods failed to detect carbapenemase production in these 6 ceftazidime-avibactam-resistant isolates, and they showed *in vitro* susceptibility to imipenem and meropenem. All of them rendered positive results for *bla*_KPC_ by PCR, and amplicon sequencing identified *bla*_KPC-31_ variant in 5 isolates and a novel variant, named *bla*_KPC-115_, in the other. Moreover, matrix-assisted laser desorption ionization–time of flight mass spectrometry was able to detect KPC in all isolates. Ceftazidime-avibactam-resistant isolates, as well as those recovered from previous infection episodes (KPC-3-producing K. pneumoniae, ceftazidime-avibactam susceptible), displayed a unique pulse type and belonged to ST11. Based on whole-genome sequencing results of selected isolates, less than 7 single-nucleotide polymorphisms were identified among them, which was indicative of the presence of a unique clone. Both *in vivo* selection and horizontal transmission seemed to have occurred in our hospital. Detection of these strains is challenging for the laboratory. History of previous KPC-E infections or colonization and systematic testing for resistance to ceftazidime-avibactam might help raise awareness of this possibility.

**IMPORTANCE** Klebsiella pneumoniae is one of the main bacteria that cause infections in health care settings. This pathogen has developed a high level of resistance to many antibiotics. Some K. pneumoniae isolates can produce an enzyme known as carbapenemase KPC, making carbapenems (considered the last line for therapy) not effective to treat their infections. The combination ceftazidime-avibactam, approved by FDA in 2015, is useful to treat infections caused by KPC-producing K. pneumoniae. This study describes the emergence, in one hospital in Argentina, of K. pneumoniae isolates that produce KPC variants (KPC-31 and KPC-115) resistant to ceftazidime-avibactam. The ceftazidime-avibactam-resistant bacteria were isolated in inpatients, including some that previously received this combination as treatment. Transmission of this strain to other patients also occurred in the studied period. Detection of these bacteria is challenging for the laboratory. The knowledge and awareness of the emergence of this pathogen in our region are highly valuable.

## INTRODUCTION

Resistance to carbapenems in *Enterobacterales*, mainly associated with carbapenemase production, has significantly increased worldwide in the last decade (1, 2). Although carbapenemases include a wide range of β-lactamase families, KPC, NDM, and OXA are the most prevalent (2–5). Infections caused by carbapenemase-producing *Enterobacterales* (CPE) have limited antimicrobial options (1, 6). In recent years, new β-lactam/β-lactamase combinations, such as ceftazidime-avibactam, meropenem-vaborbactam, and imipenem-relebactam, have been introduced into clinical use displaying excellent activity against class A carbapenemase producers (1, 7, 8). The Infectious Diseases Society of America has recently recommended these new combinations for the treatment of severe CPE infections (9). Ceftazidime-avibactam was the first of these new combinations to be introduced for the treatment of KPC-producing *Enterobacterales* (KPC-E). Not surprisingly, ceftazidime-avibactam resistance was described soon after its introduction in KPC-producing Klebsiella pneumoniae isolates (KPC-Kp) (10–12). Resistance to ceftazidime-avibactam in KPC-E, still reported at very low rates in different studies, may be mediated by several mechanisms, such as missense mutations or deletions in the Ω-loop of the carbapenemase or permeability defects coupled with augmented β-lactamase expression (12–15). At present, more than 100 variants of KPC carbapenemase have been reported, some of them associated with resistance to ceftazidime-avibactam.

In this study, we describe an outbreak in an intensive care unit (ICU) by K. pneumoniae sequence type 11 (ST11) producing KPC-31 and KPC-115. These bacteria displayed resistance to ceftazidime-avibactam, but *in vitro* susceptibility to meropenem or imipenem was observed. To our knowledge, neither KPC-31 nor the novel variant KPC-115 has been previously described in Argentina or even in Latin America.

## RESULTS

The patients herein included underwent the usual long-term hospitalization at an ICU described worldwide for severe COVID pneumonia, followed by several hospital-acquired infections requiring different antimicrobial treatments. Fecal colonization and even infections by KPC-Kp occurred in these patients, and ceftazidime-avibactam was used as part of the treatment in 3 of them. Patients’ main epidemiological characteristics, as well as antibiotic treatments and outcomes, are summarized in Table 1. Three patients had bacteremia (patients 3, 5, and 6), 1 urinary tract infection (patient 4), and 2 had no infection (patients 1 and 2) but colonization in the tracheal aspirate and central venous catheter tip.

**TABLE 1:** Patients’ clinical features^*a*^

Patient	Gender/age	Date of hospitalization	Severe COVID pneumonia	Positive rectal cultures for KPC-E	Infection with Kp-KPC in preceding mo	Previous use of CZA	Main treatment for the CZA-resistant episode	Outcome
1	M/53	03-21-2021	Y	Y	Y	Y	No atb. catheter removal	Discharge
2	M/47	05-27-2021	Y	Y	N	N	No atb. colonization	Discharge
3	F/71	05-28-2021	Y	Y	N	N	MEM + AMK	Discharge
4	M/46	05-22-2021	Y	Y	N	N	AMK	Discharge
5	F/36	05-21-2021	Y	Y	Y	Y	MEM + AMK	Death^*b*^
6	M/73	06-07-2021	N	Y	Y	Y	CZA + AMK, then MEM + AMK + FOF	Discharge

a F, female; M, male; Y, yes; N, no; CZA, ceftazidime-avibactam; No atb., no antibiotics were administered for these episodes because either removal of catheter or colonization was assumed; MEM, meropenem; AMK, amikacin; FOF, fosfomycin; KPC-E, KPC-producing *Enterobacterales*; KPC-Kp, KPC-producing Klebsiella pneumoniae. Both KPC-E colonization and KPC-Kp were considered in the preceding month of CZA-resistant isolation.

b Death occurred more than 30 days after CZA-resistant infection, being nonrelated to it.

The features of K. pneumoniae isolates, antimicrobial susceptibilities, and carbapenemase detection are outlined in Table 2. Six out of 15 isolates displayed resistance to ceftazidime-avibactam but susceptibility to imipenem and meropenem, while the remaining 9 were susceptible to ceftazidime-avibactam and resistant to imipenem and meropenem. All isolates were resistant to colistin, trimethoprim-sulfamethoxazole, and ciprofloxacin but susceptible to gentamicin, amikacin, and fosfomycin. MIC values for tigecycline were 2 to 4 mg/L (susceptible/intermediate). Ceftazidime-avibactam-resistant K. pneumoniae isolates rendered negative results for carbapenemase production in the double disc synergy test, BlueCarba assay, and modified Hodge test. Among the latest, lateral flow immunochromatographic assay (LFIA) was only positive in K. pneumoniae 5F. The matrix-assisted laser desorption ionization–time of flight mass spectrometry (MALDI-TOF MS)-based procedure was able to detect KPC in all isolates, including those resistant to ceftazidime-avibactam. Accordingly, multiplex PCR confirmed *bla*_KPC_ presence and ruled out other common carbapenemase genes, such as *bla*_NDM_, *bla*_VIM_, *bla*_IMP_, or *bla*_OXA-group-48_. The 9 ceftazidime-avibactam-susceptible isolates presented *bla*_KPC-3_. On the other hand, in the ceftazidime-avibactam-resistant isolates, *bla*_KPC-31_ was detected in 5 isolates and a novel variant called *bla*_KPC-115_ (GenBank accession number OM714909) in the remaining 1 isolate (Table 2). This new variant displayed a 6-nucleotide deletion translated into a 2-amino-acid deletion (absence of Asp169 and Ser170) and 1 substitution (Leu168Pro) compared to KPC-3.

**TABLE 2:** Bacterial features, β-lactam susceptibilities, results of carbapenemase-detection methods, and KPC variants^*a*^

Patient	Isolate	2021 date (mo-day)	Source^*b*^	Antimicrobial susceptibility^*c*^ (MIC in mg/L/interpretation)										KPC detection^*d*^			KPC-variant
														DD-PBA, BCA, MHT	LFIA	MALDI-TOF MS	
				CZA	MEM	IPM	ETP	FEP	CAZ	CRO	TZP	SAM	FOX				
1	1A	04-04	R	1/S	>32/R	>8/R	>1/R	>16/R	>16/R	>4/R	>64/R	>16/R	>16/R	+	+	+	3
	1B	04-04	U	1/S	>32/R	>8/R	>1/R	>16/R	>16/R	>4/R	>64/R	>16/R	>16/R	+	+	+	3
	**1C**	**04-15**	**C**	**>256/R**	**≤0.5/S**	**≤0.25/S**	**>1/R**	**16/R**	**>16/R**	**>4/R**	**16/S**	**16/I**	**≤4/S**	**–**	**–**	**+**	**31**
2	**2A**	**06-06**	**R**	**>256/R**	**≤0.5/S**	**≤0.25/S**	**>1/R**	**16/R**	**>16/R**	**>4/R**	**2/S**	**16/I**	**≤4/S**	**–**	**–**	**+**	**31**
3	**3A**	**06-08**	**B**	**>256/R**	**≤0.5/S**	**1/S**	**>1/R**	**16/R**	**>16/R**	**>4/R**	**8/S**	**16/I**	**≤4/S**	**–**	**–**	**+**	**31**
4	**4A**	**06-24**	**U**	**>256/R**	**≤0.5/S**	**≤0.25/S**	**>1/R**	**16/R**	**>16/R**	**>4/R**	**2/S**	**16/I**	**8/S**	**–**	**–**	**+**	**31**
5	5A	06-20	C	1/S	>32/R	>8/R	>1/R	>16/R	>16/R	>4/R	>64/R	>16/R	>16/R	+	+	+	3
	5B	06-25	R	1/S	>32/R	>8/R	>1/R	>16/R	>16/R	>4/R	>64/R	>16/R	>16/R	+	+	+	3
	5C	07-03	R	1/S	>32/R	>8/R	>1/R	>16/R	>16/R	>4/R	>64/R	>16/R	>16/R	+	+	+	3
	5D	07-03	C	1/S	8/R	8/R	>1/R	>16/R	>16/R	>4/R	>64/R	>16/R	16/I	+	+	+	3
	5E	07-03	B	1/S	8/R	8/R	>1/R	>16/R	>16/R	>4/R	>64/R	>16/R	16/I	+	+	+	3
	**5F**	**07-15**	**B**	**24/R**	**≤0.5/S**	**≤0.25/S**	**0.5/S**	**2/S**	**>16/R**	**>4/R**	**≤4/S**	**>16/R**	**≤4/S**	**–**	**+**	**+**	**115**
6	6A	06-19	R	2/S	>32/R	>8/R	>1/R	>16/R	>16/R	>4/R	>64/R	>16/R	>16/R	+	+	+	3
	6B	06-25	B	2/S	>32/R	>8/R	>1/R	>16/R	>16/R	>4/R	>64/R	>16/R	>16/R	+	+	+	3
	**6C**	**07-20**	**B**	**64/R**	**≤0.5/S**	**≤0.25/S**	**>1/R**	**16/R**	**>16/R**	**>4/R**	**8/S**	**>16/R**	**≤4/S**	**–**	**–**	**+**	**31**

a Ceftazidime-avibactam-resistant isolates are shown in bold.

b Sources: R, respiratory; U, urine; C, catheter; B, blood.

c Antimicrobials: CZA, ceftazidime-avibactam; MEM, meropenem; IPM, imipenem; ETP, ertapenem; FEP, cefepime; CAZ, ceftazidime; CRO, ceftriaxone; TZP, piperacillin-tazobactam; SAM, ampicillin-sulbactam; FOX, cefoxitin.

d KPC detection: DD-PBA, double disk diffusion with phenyl-boronic acid; BCA, BlueCarba assay; MHT, modified Hodge test; LFIA, lateral flow immunoassay; MALDI-TOF MS, use of this technology for detection of mature KPC protein.

All isolates displayed a unique pulse type and belonged to ST11, regardless of the KPC variant and ceftazidime-avibactam or carbapenem susceptibilities. In all isolates, the genetic context of *bla*_KPC_ belonged to Tn*4401a*. Based on WGS results, isolates 1A, 1C, 5B, and 5F belonged to ST11 and presented less than 7 single-nucleotide polymorphisms (SNPs) among them, corresponding to a unique clone. Moreover, isolates recovered from the same patient, 1A (ceftazidime-avibactam-susceptible) versus 1C (ceftazidime-avibactam-resistant), displayed 3 SNPs, while 5B (ceftazidime-avibactam-susceptible) versus 5F (ceftazidime-avibactam-resistant) displayed 4 SNPs.

In furtherance, the same acquired markers responsible for quinolone resistance [GyrA-83I, GyrA-87N, ParC-80I, *OqxA*, *OqxB*, *aac(6′)-Ib-cr*], aminoglycosides [*aac(6′)-Ib-cr*, *aadA2*, *aadA*, *aph3-Ia*], and sulfonamides (*sul1*, *sul3*) were detected in all genomes. With respect to colistin resistance, plasmid-encoded *mcr* markers were not detected in any isolate. Moreover, wild-type alleles were observed in *mgrB* and *pmrCAB* operons. However, PhoP displayed 1 substitution (Arg34Lys) and in PhoQ multiple substitutions were observed (Lys64Arg, Lys92Gln, Thr106Ala, Asp112Glu, Val139Ile, Phe163Leu, Ile198Val, Ser372Thr, Pro424Gln, Leu482Gln, and Glu487Gln). These findings will be the subject of future studies. The capsular *loci* belonged to *wzi* 39, K_Locus KL39, and O_Locus O3b. The Yersiniabactin coding gene in ICEKp10 (YbST: 22-2LV) and colibactin *clb3* (CbST: 13-2LV) were detected.

## DISCUSSION

We report on an outbreak of KPC-Kp ST11 in the ICU of a private university hospital in Buenos Aires, Argentina, during the COVID pandemic. This episode was characterized by the emergence of ceftazidime-avibactam-resistant isolates. *In vivo* selection of these isolates was inferred in 3 patients (patients 1, 5, and 6) after ceftazidime-avibactam use. This resistant profile was mediated by modifications in KPC-3 (found in previous isolates in these patients), which resulted in KPC-31 and the novel variant KPC-115. Recently, Arcari et al. (16) described an interplay between KPC-3 and KPC-31 in patients with COVID that were treated with ceftazidime-avibactam, which was similar to the situation observed in our hospital. Additionally, some ceftazidime-avibactam-resistant isolates were recovered from patients where ceftazidime-avibactam was never used (patients 2, 3, and 4). We could therefore infer that interpatient transmission also occurred. These findings are consistent with previous reports (14).

Upon outbreak suspicion, the ICU was placed on epidemiologic alert to reinforce infection control measures for carbapenemases carriage and restrict the use of ceftazidime-avibactam. These actions succeeded in containing the outbreak, since no other KPC-producing bacteria resistant to ceftazidime-avibactam were detected after July 2021. Infection episodes were treated with antibiotics with apparent *in vitro* activity according to their MIC, such as meropenem and amikacin. No clinical or microbiological failures were noted, which is consistent with previous similar reports (14, 16–19). Patients colonized with ceftazidime-avibactam-resistant isolates (patients 1 and 2) received no antibiotic treatment for these instances.

The detection and report of these ceftazidime-avibactam-resistant KPC variants present a great challenge. As previously described, these isolates mostly appear “susceptible” to imipenem and meropenem, showing an extended-spectrum β-lactamase-like phenotype (20–22). Moreover, isolates showed resistance to ceftazidime-avibactam, a phenotype common for metalo-carbapenemase producers, which was ruled out due to negative results in both EDTA synergy test and multiplex PCR. A determined effort was made to elucidate the resistance mechanism due to atypical ertapenem nonsusceptibility and colistin resistance (mostly seen in KPC-Kp in our facility), as well as history of colonization or infection with KPC-Kp. In addition, and in accordance with previous studies, conventional phenotypic methods failed to detect the presence of KPC-31 carbapenemase (20–22), as well as the new variant KPC-115 (Table 2). Immunochromatographic detection carried out on ceftazidime-avibactam-resistant isolates allowed to detect KPC-115 but not KPC-31 producers (Table 2). Antonelli et al. (20) have already described failure of LFIA to detect KPC-31. This was also confirmed by other articles, although detection of other KPC variants showing ceftazidime-avibactam resistance was reported (17, 22). An exhaustive bibliographic revision of ceftazidime-avibactam-resistant KPC variants evidenced that there is no correlation among Ω-loop mutations and the LFIA detection. On the other hand, specific peak identification by MALDI-TOF MS contributed to the detection of all KPC variants, both KPC-31 and KPC-115 in those ceftazidime-avibactam-resistant isolates, as well as KPC-3 in ceftazidime-avibactam-susceptible isolates (Table 2). Therefore, this method, when available, could prove useful in clinical practice to directly identify different KPC variants (23). These results were further confirmed by PCR amplification and sequencing.

It is also important to comment that isolates producing KPC variants with ceftazidime-avibactam resistance might not grow in chromogenic media for detection of CPE fecal colonization because of their low carbapenem MIC. In fact, although not systematically investigated, some of our patients rendered rectal swabs negative for KPC producers at the time they were infected with KPC-31 (data not shown).

Although ceftazidime-avibactam-resistant K. pneumoniae isolates belonging to ST11 were previously reported in Argentina, they produced KPC-8 displaying enhanced catalytic efficiency toward ceftazidime (24). KPC-31 presents a single point mutation (Asp179Tyr) in the Ω-loop, assumed to be responsible for ceftazidime-avibactam resistance and restoration of the *in vitro* susceptibility to imipenem and meropenem (21). It was postulated that modifications in the Ω-loop drive to a higher affinity to ceftazidime, leading to ceftazidime-avibactam resistance, although with a shortfall in the hydrolytic efficiency on other β-lactams, such as carbapenems, cefotaxime, and piperacillin, among others. This hydrolytic profile may be partially observed in our results (Table 2) since MIC values for imipenem, meropenem, piperacillin-tazobactam, and cefoxitin for ceftazidime-avibactam-resistant isolates were lower than those for ceftazidime-avibactam-susceptible ones.

KPC-31 was described in some European countries and the United States (20, 21), while this report is the first to describe KPC-31 in Latin America.

KPC-115 displayed a 2-point deletion and 2 mutations compared to KPC-3, all located in the Ω-loop. Antinori et al. (25) also reported a KPC-3 variant with a 2-amino-acid deletion in the Ω-loop (glutamic acid and leucine in positions 167 and 168), near that observed in KPC-115. This KPC variant, as well as KPC-115, was resistant to ceftazidime-avibactam, susceptible to imipenem and meropenem, and rendered a positive result with LFIA (25). Although kinetic studies are required, these mutations are probably associated with an enhanced affinity toward ceftazidime, prevention of binding to avibactam, and reversion of carbapenem resistance, as previously reported for other variants (15, 26).

## MATERIALS AND METHODS

### Patient characteristics, bacterial isolates, and antimicrobial susceptibility.

Ceftazidime-avibactam-resistant K. pneumoniae isolates were found in 6 patients hospitalized at the ICU of our institution in the city of Buenos Aires between April and July 2021. All these patients presented several comorbidities and required mechanical ventilation, urinary catheters, and central venous catheters, and 5 of them were admitted because of severe COVID pneumonia. Main clinical information was collected from medical records (Table 1). Fecal colonization with CPE was assessed by rectal swabs seeded onto CHROMAgar KPC medium (CHROMagar, France) followed by phenotypic and molecular carbapenemase characterization of suspected colonies. Isolates from fecal colonization were not included because they were not available for further investigation. All K. pneumoniae isolates (*n* = 15) recovered from clinical samples of these inpatients were included in this study. Isolates were identified by MALDI-TOF MS (Becton, Dickinson-Bruker Daltonics Biotyper, USA). Ceftazidime-avibactam MIC was determined by the epsilometric method (Liofilchem, Italy), colistin MIC by broth microdilution (SensiTest Colistin, Liofilchem, Italy), fosfomycin MIC by agar dilution, and susceptibilities to other antimicrobials by Phoenix automatic system (Becton, Dickinson, USA). Susceptibility categories were interpreted according to EUCAST for colistin and fosfomycin, FDA for tigecycline, and CLSI for the remaining antibiotics (27–29).

### Carbapenemase characterization.

The presence of carbapenemases was analyzed by phenotypic procedures, including a double disc inhibition test using both phenyl-boronic acid and EDTA, as well as Blue-Carba assay, modified Hodge test, LFIA (NG-Test CARBA 5; NG Biotech, France), and a MALDI-TOF MS-based procedure to detect mature KPC protein (23).

Genotypic detection of carbapenemase coding genes was assessed by multiplex PCR for *bla*_KPC_, *bla*_OXA__-__48 group_, *bla*_NDM_, *bla*_IMP_, and *bla*_VIM_ (30). Complete *bla*_KPC_ was amplified as previously described (31), and its genetic context was studied by a PCR mapping approach (32). Amplicons were sequenced at external facilities (Macrogen Inc., South Korea) and analyzed using the BLAST tool of the National Center for Biotechnology Information.

### Molecular characterization of KPC-producing K. pneumoniae isolates.

Clonal relationship was analyzed by pulsed-field gel electrophoresis after digestion of genomic DNA with XbaI (XbaI-PFGE) (33). Additionally, WGS was performed in 4 selected isolates (1A, 1C, 5B, and 5F). Bacterial DNA was extracted using QIAamp DNA minikit (Qiagen, Germany) from overnight cultures and further underwent WGS with the Illumina MiSeq platform (Illumina Inc., USA), using a 2× 150-bp paired-end approach. *De novo* assemblies of WGS data were generated using SPAdes software v3.13.3 (34). The ST was investigated utilizing the “Klebsiella pneumoniae” database PubMLST. Antimicrobial resistance genes were studied using ResFinder 4.0 (35), and K-type and virulence factors were studied using Kleborate v2.2.0 (36). Phylogeny was inferred using CSI Phylogeny 1.4 and visualized using FigTree 1.3.1 (37). SNPs were analyzed on the whole-genome by CSI Phylogeny 1.4 within the following parameters: select minimum depth at SNP position at 10×, minimum distance between SNPs at 10 bp, and minimum SNP quality score of 30 (37).

### Data availability.

The genomes of K. pneumoniae 1A, 1C, 5B, and 5F have been deposited at GenBank under Bioproject PRJNA810071 and accession number SAMN26232644, SAMN26232645, SAMN26232646, and SAMN26232647, respectively.

The sequence of the novel *bla*_KPC-115_ has been deposited at GenBank under accession number OM714909.1.

## References

[1] Bonomo RA, Burd EM, Conly J, Limbago BM, Poirel L, Segre JA, Westblade LF. 2018. Carbapenemase-producing organisms: a global scourge. Clin Infect Dis 66:1290–1297. doi:10.1093/cid/cix893. 29165604 PMC5884739

[2] Bush K, Bradford PA. 2020. Epidemiology of β-lactamase-producing pathogens. Clin Microbiol Rev 33:e00047-19. doi:10.1128/CMR.00047-19. 32102899 PMC7048014

[3] Brink AJ. 2019. Epidemiology of carbapenem-resistant Gram-negative infections globally. Curr Opin Infect Dis 32:609–616. doi:10.1097/QCO.0000000000000608. 31567571

[4] Logan LK, Weinstein RA. 2017. The epidemiology of carbapenem-resistant Enterobacteriaceae: the impact and evolution of a global menace. J Infect Dis 215:S28–S36. doi:10.1093/infdis/jiw282. 28375512 PMC5853342

[5] Queenan AM, Bush K. 2007. Carbapenemases: the versatile β-lactamases. Clin Microbiol Rev 20:440–458. doi:10.1128/CMR.00001-07. 17630334 PMC1932750

[6] Perez F, El Chakhtoura NG, Papp-Wallace KM, Wilson BM, Bonomo RA. 2016. Treatment options for infections caused by carbapenem-resistant Enterobacteriaceae: can we apply “precision medicine” to antimicrobial chemotherapy? Expert Opin Pharmacother 17:761–781. doi:10.1517/14656566.2016.1145658. 26799840 PMC4970584

[7] Yahav D, Giske CG, Grāmatniece A, Abodakpi H, Tam VH, Leibovici L. 2020. New β-lactam–β-lactamase inhibitor combinations. Clin Microbiol Rev 34:e00115-20. doi:10.1128/CMR.00115-20. PMC7667665 33177185

[8] Zasowski EJ, Rybak JM, Rybak MJ. 2015. The β-lactams strike back: ceftazidime-avibactam. Pharmacother 35:755–770. doi:10.1002/phar.1622. PMC4545577 26289307

[9] Tamma P, Aitken S, Bonomo R, Mathers AJ, van Duin D, Clancy CJ. 2022. IDSA guidance on the treatment of antimicrobial-resistant gram-negative infections: version 1.0. Infectious Diseases Society of America. https://www.idsociety.org/practice-guideline/amr-guidance/#. Accessed 8 April 2022. 10.1093/cid/ciad428 37463564

[10] Humphries RM, Yang S, Hemarajata P, Ward KW, Hindler JA, Miller SA, Gregson A. 2015. First report of ceftazidime-avibactam resistance in a KPC-3-expressing *Klebsiella pneumoniae* isolate. Antimicrob Agents Chemother 59:6605–6607. doi:10.1128/AAC.01165-15. 26195508 PMC4576121

[11] Shields RK, Chen L, Cheng S, Chavda KD, Press EG, Snyder A, Pandey R, Doi Y, Kreiswirth BN, Nguyen MH, Clancy CJ. 2017. Emergence of ceftazidime-avibactam resistance due to plasmid-borne *bla*_KPC-3_ mutations during treatment of carbapenem-resistant *Klebsiella pneumoniae* infections. Antimicrob Agents Chemother 61:e02097-16. doi:10.1128/AAC.02097-16. 28031201 PMC5328542

[12] Zhang P, Shi Q, Hu H, Hong B, Wu X, Du X, Akova M, Yu Y. 2020. Emergence of ceftazidime/avibactam resistance in carbapenem-resistant *Klebsiella pneumoniae* in China. Clin Microbiol Infect 26:124.e1–124.e4. doi:10.1016/j.cmi.2019.08.020. 31494252

[13] de Jonge BL, Karlowsky JA, Kazmierczak KM, Biedenbach DJ, Sahm DF, Nichols WW. 2016. In vitro susceptibility to ceftazidime-avibactam of carbapenem-nonsusceptible Enterobacteriaceae isolates collected during the INFORM Global Surveillance Study (2012 to 2014). Antimicrob Agents Chemother 60:3163–3169. doi:10.1128/AAC.03042-15. 26926648 PMC4862516

[14] Di Bella S, Giacobbe DR, Maraolo AE, Viaggi V, Luzzati R, Bassetti M, Luzzaro F, Principe L. 2021. Resistance to ceftazidime/avibactam in infections and colonisations by KPC-producing *Enterobacterales*: a systematic review of observational clinical studies. J Glob Antimicrob Resist 25:268–281. doi:10.1016/j.jgar.2021.04.001. 33895414

[15] Wang Y, Wang J, Wang R, Cai Y. 2020. Resistance to ceftazidime-avibactam and underlying mechanisms. J Glob Antimicrob Resist 22:18–27. doi:10.1016/j.jgar.2019.12.009. 31863899

[16] Arcari G, Oliva A, Sacco F, Di Lella FM, Raponi G, Tomolillo D, Curtolo A, Venditti M, Carattoli A. 2022. Interplay between *Klebsiella pneumoniae* producing KPC-31 and KPC-3 under treatment with high dosage meropenem: a case report. Eur J Clin Microbiol Infect Dis 41:495–500. doi:10.1007/s10096-021-04388-y. 34988712 PMC8731190

[17] Cano Á, Guzmán-Puche J, García-Gutiérrez M, Castón JJ, Gracia-Ahufinger I, Pérez-Nadales E, Recio M, Natera AM, Marfil-Pérez E, Martínez-Martínez L, Torre-Cisneros J. 2020. Use of carbapenems in the combined treatment of emerging ceftazidime-avibactam-resistant and carbapenem-susceptible KPC-producing *Klebsiella pneumoniae* infections: report of a case and review of the literature. J Glob Antimicrob Resist 22:9–12. doi:10.1016/j.jgar.2019.11.007. 31733412

[18] Shields RK, Nguyen MH, Press EG, Chen L, Kreiswirth BN, Clancy CJ. 2017. Emergence of ceftazidime-avibactam resistance and restoration of carbapenem susceptibility in *Klebsiella pneumoniae* carbapenemase-producing *K pneumoniae*: a case report and review of literature. Open Forum Infect Dis 4:ofx101. doi:10.1093/ofid/ofx101. 28685153 PMC5493938

[19] Shields RK, Nguyen MH, Press EG, Chen L, Kreiswirth BN, Clancy CJ. 2017. *In vitro* selection of meropenem resistance among ceftazidime-avibactam-resistant, meropenem-susceptible *Klebsiella pneumoniae* isolates with variant KPC-3 carbapenemases. Antimicrob Agents Chemother 24:e00079-17. doi:10.1128/aac.00079-17. PMC5404588 28242667

[20] Antonelli A, Giani T, Di Pilato V, Riccobono E, Perriello G, Mencacci A, Rossolini GM. 2019. KPC-31 expressed in a ceftazidime-avibactam-resistant *Klebsiella pneumoniae* is associated with relevant detection issues. J Antimicrob Chemother 74:2464–2466. doi:10.1093/jac/dkz156. 31318973

[21] Haidar G, Clancy CJ, Shields RK, Hao B, Cheng S, Nguyen MH. 2017. Mutations in *bla*KPC-3 that confer ceftazidime-avibactam resistance encode novel KPC-3 variants that function as extended-spectrum β-lactamases. Antimicrob Agents Chemother 61:e02534-16. doi:10.1128/AAC.02534-16. 28223379 PMC5404534

[22] Bianco G, Boattini M, Iannaccone M, Bondi A, Ghibaudo D, Zanotto E, Peradotto M, Cavallo R, Costa C. 2021. Carbapenemase detection testing in the era of ceftazidime/avibactam-resistant KPC-producing *Enterobacterales*: a 2-year experience. J Glob Antimicrob Resist 24:411–414. doi:10.1016/j.jgar.2021.02.008. 33621692

[23] Figueroa-Espinosa R, Costa A, Cejas D, Barrios R, Vay C, Radice M, Gutkind G, Di Conza J. 2019. MALDI-TOF MS based procedure to detect KPC-2 directly from positive blood culture bottles and colonies. J Microbiol Methods 159:120–127. doi:10.1016/j.mimet.2019.02.020. 30849422

[24] García J, Nastro M, Cejas D, Santana G, Mancino MB, Hidalgo M, Maccallini G, Vay C, Radice M, Dabos L, Famiglietti A, Rodríguez H. 2020. Emergence of ceftazidime/avibactam resistance in KPC-8 producing *Klebsiella pneumoniae* in South America. Clin Microbiol Infect 26:1264–1265. doi:10.1016/j.cmi.2020.03.013. 32217161

[25] Antinori E, Unali I, Bertoncelli A, Mazzariol A. 2020. *Klebsiella pneumoniae* carbapenemase (KPC) producer resistant to ceftazidime-avibactam due to a deletion in the *bla*KPC3 gene. Clin Microbiol Infect 26:946.e1–946.e3. doi:10.1016/j.cmi.2020.02.007. 32061796

[26] Winkler ML, Papp-Wallace KM, Bonomo RA. 2015. Activity of ceftazidime/avibactam against isogenic strains of *Escherichia coli* containing KPC and SHV β-lactamases with single amino acid substitutions in the Ω-loop. J Antimicrob Chemother 70:2279–2286. doi:10.1093/jac/dkv094. 25957381 PMC4500773

[27] Clinical and Laboratory Standards Institute. 2022. CLSI performance standards for antimicrobial susceptibility testing. 32st ed. CLSI supplement M100. Clinical and Laboratory Standards Institute, Wayne, PA.

[28] FDA. Antibacterial susceptibility test interpretive criteria. 2022. https://www.fda.gov/drugs/development-resources/tigecycline-injection-products. Accessed 8 April 2022.

[29] The European Committee on Antimicrobial Susceptibility Testing. 2022. Breakpoint tables for interpretation of MICs and zone diameters: version 12.0. http://www.eucast.org. Accessed 8 April 2022.

[30] Poirel L, Walsh TR, Cuvillier V, Nordmann P. 2011. Multiplex PCR for detection of acquired carbapenemase genes. Diagn Microbiol Infect Dis 70:119–123. doi:10.1016/j.diagmicrobio.2010.12.002. 21398074

[31] Bradford PA, Bratu S, Urban C, Visalli M, Mariano N, Landman D, Rahal JJ, Brooks S, Cebular S, Quale J. 2004. Emergence of carbapenem-resistant *Klebsiella* species possessing the class A carbapenem-hydrolyzing KPC-2 and inhibitor-resistant TEM-30 β-lactamases in New York City. Clin Infect Dis 39:55–60. doi:10.1086/421495. 15206053

[32] Naas T, Cuzon G, Villegas MV, Lartigue MF, Quinn JP, Nordmann P. 2008. Genetic structures at the origin of acquisition of the β-lactamase *bla*_KPC_ gene. Antimicrob Agents Chemother 52:1257–1263. doi:10.1128/AAC.01451-07. 18227185 PMC2292522

[33] Miranda G, Kelly C, Solorzano F, Leanos B, Coria R, Patterson JE. 1996. Use of pulsed-field gel electrophoresis typing to study an outbreak of infection due to *Serratia marcescens* in a neonatal intensive care unit. J Clin Microbiol 34:3138–3141. doi:10.1128/jcm.34.12.3138-3141.1996. 8940460 PMC229471

[34] Prjibelski A, Antipov D, Meleshko D, Lapidus A, Korobeynikov A. 2020. Using SPAdes de novo assembler. Curr Protoc Bioinformatics 70:e102. doi:10.1002/cpbi.102. 32559359

[35] Bortolaia V, Kaas RS, Ruppe E, Roberts MC, Schwarz S, Cattoir V, Philippon A, Allesoe RL, Rebelo AR, Florensa AF, Fagelhauer L, Chakraborty T, Neumann B, Werner G, Bender JK, Stingl K, Nguyen M, Coppens J, Xavier BB, Malhotra-Kumar S, Westh H, Pinholt M, Anjum MF, Duggett NA, Kempf I, Nykäsenoja S, Olkkola S, Wieczorek K, Amaro A, Clemente L, Mossong J, Losch S, Ragimbeau C, Lund O, Aarestrup FM. 2020. ResFinder 4.0 for predictions of phenotypes from genotypes. J Antimicrob Chemother 75:3491–3500. doi:10.1093/jac/dkaa345. 32780112 PMC7662176

[36] Lam MMC, Wick RR, Watts SC, Cerdeira LT, Wyres KL, Holt KE. 2021. A genomic surveillance framework and genotyping tool for *Klebsiella pneumoniae* and its related species complex. Nat Commun 12:4188. doi:10.1038/s41467-021-24448-3. 34234121 PMC8263825

[37] Kaas RS, Leekitcharoenphon P, Aarestrup FM, Lund O. 2014. Solving the problem of comparing whole bacterial genomes across different sequencing platforms. PLoS One 9:e104984. doi:10.1371/journal.pone.0104984. 25110940 PMC4128722

